# Quantifying Vascular Density in Tissue Engineered Constructs Using Machine Learning

**DOI:** 10.3389/fphys.2021.650714

**Published:** 2021-04-27

**Authors:** Hannah A. Strobel, Alex Schultz, Sarah M. Moss, Rob Eli, James B. Hoying

**Affiliations:** ^1^Tissue Modeling, Advanced Solutions Life Sciences, Manchester, NH, United States; ^2^Innovations Laboratory, Advanced Solutions Life Sciences, Louisville, KY, United States

**Keywords:** angiogenesis, machine learning, vascularity, neovessel growth, vessel quantification

## Abstract

Given the considerable research efforts in understanding and manipulating the vasculature in tissue health and function, making effective measurements of vascular density is critical for a variety of biomedical applications. However, because the vasculature is a heterogeneous collection of vessel segments, arranged in a complex three-dimensional architecture, which is dynamic in form and function, it is difficult to effectively measure. Here, we developed a semi-automated method that leverages machine learning to identify and quantify vascular metrics in an angiogenesis model imaged with different modalities. This software, BioSegment, is designed to make high throughput vascular density measurements of fluorescent or phase contrast images. Furthermore, the rapidity of assessments makes it an ideal tool for incorporation in tissue manufacturing workflows, where engineered tissue constructs may require frequent monitoring, to ensure that vascular growth benchmarks are met.

## Introduction

A mature vascular network is essential for the viability of tissues, native or fabricated. Thus, making informative, quantitative measurements of a vascular network is important. This is especially relevant for a maturing, vascularized engineered tissue, which needs to meet certain vascular density benchmarks to remain viable. Making accurate vessel density measurements, particularly during angiogenesis, can be challenging due to the irregular features of both the individual vessels and the complex networks they form, and the surrounding tissue environments. Vasculatures exist in three dimensional environments with vessels extending across all dimensions, may vary in density, and can resemble other non-vascular tissue elements (e.g., ducts, cell bundles, etc.). A variety of imaging modalities are employed to visualize the vasculature in both the laboratory and the clinic (Spaide et al., 2015; Grüneboom et al., 2019; Bautista et al., 2020). Usually, a contrast agent that fills the blood space and/or labels vessel cells directly is involved, as inherent contrast between the vessel and the surrounding tissue is often low. Segmentation and quantification from these images is subsequently performed to assess the vasculature. While effective at visualizing vessel elements, non-uniform labeling of vessels by these agents can confound segmentation and feature detection. All of this complicates computer-based image analyses. Additionally, many labeling methods rely on contrast agents flowing through the vasculature and are, therefore, not useable for neovascular systems in which intravascular perfusion is not yet established.

Other pixel-based computational systems for quantifying do exist, designed to analyze images of vasculatures stained with fluorescent labels, but are often intended for very specific applications. The ImageJ plugin Angiogenesis Analyzer, for example, has been widely published for use in 2D endothelial cell assays (Yamamoto et al., 2014; Yang et al., 2016) and a handful of 3D tissues where the vasculature was very distinct and uniform (Samarelli et al., 2014). Rarely, however, in tissues, fabricated or native, is the vasculature so clear. Often clumps of cells or tissue, or irregularly shaped immature vessels, present challenges for computational methods of identifying blood vessels, even with highly specific stains.

Artificial intelligence and machine learning (AI/ML) are increasingly employed to make measurements in biomedical research that can be challenging for more traditional computational systems biological systems. With AI/ML, a software program can be “trained” to distinguish certain features. A handful of AI/ML programs exist that have been used to quantify the vasculature, although none have been designed specifically for high throughput analyses, especially involving phase contrast imaging. Additionally, all of these programs have only been tested on clearly defined, mature vasculatures, that lack the feature noise visible in growing and remodeling neovasculatures in a tissue space. For example, the program VesSAP has been used to map the vasculature in a whole mouse brain following the use of a perfused tag. While the program was able to accurately map the vasculature and produce an impressive amount of data, extensive clearing and staining protocols were needed to obtain clean, high resolution 3D images, which took an additional 24 h to segment (Todorov et al., 2020). While this may be ideal for some applications, it may not be as useful for high throughput analysis or as a routine-use tool in the laboratory. VesSAP also requires a background in computer software to operate. Another program, REAVER, has been used in similar applications, but also requires a MATLAB license and an understanding of software coding in order to use (Corliss et al., 2020). The open source software Ilastik has been used to quantify vascular density using ML (Bochner et al., 2020). However, it also can be challenging to use by the non-expert and requires more computing power to run than is available to a typical biomedical laboratory.

Perhaps because existing programs can be challenging to use, and are designed for very specific applications, the most common approach for measuring vessel density is still having an expert user manually trace vessels in each individual image (Holley et al., 2010; Top et al., 2011; Da et al., 2015; Cossutta et al., 2019). While manual annotations are an easy way to accurately quantify vessel density, it can be very tedious and time consuming, and is not a practical way to analyze large amounts of data.

Here, we leveraged modern ML to develop an easy-to-use analysis tool specifically designed to make measurements of vascularity in *in vitro* tissue environments. The focus of this application is assessing angiogenesis (new vessel growth) in which vessel morphology and network topology is highly variable. We focused on reporting vessel length density measurements from our experiments, although it may be possible for the tool to be trained to identify other features and corresponding metrics, as well. Ultimately, BioSegment will be incorporated into high-throughput tissue manufacturing workflows to monitor vascular growth within fabricated, tissue engineered products. Here, we demonstrate the application of this tool, the BioSegment software, in assessments of vascularity from confocal fluorescence and phase contrast images.

## Methods

### Microvessel Culture

Whole, intact, microvessel fragments were isolated from adipose tissue, from either discarded human lipoaspirates or epididymal fat from male retired breeder Sprague Dawley rats (all animal procedures were approved by the Dartmouth College IACUC) and assembled into angiogenesis assays as previously described (Nunes et al., 2013; Strobel et al., 2020). Rat vessels were cultured at 60 k/ml in DMEM (Gibco) containing 20% fetal bovine serum (FBS; Thermo Fisher), 1% penicillin-streptomycin (Fisher), and 1% amphotericin B (Fisher). Human microvessels were cultured at 100 k/ml in RPMI (Corning) containing B27 (Gibco) and 50 ng/ml vascular-endothelial growth factor (VEGF; Peprotech). Assessments were made of four different treatments (Groups 1–4) promoting differing levels of angiogenesis. Experimental setups are reported in detail in Strobel et al. (2020). Briefly, for fluorescent 4× images, Group 1 contained microvessels with no additional stimuli, Group 2 was treated with vascular endothelial growth factor (VEGF), stromal cells were incorporated in Groups 3 and 4, and Group 4 was treated with a VEGF trap [R&D systems, described in Strobel et al. (2020)]. For 10× images, Group 1 contained microvessels alone, Group 2 contained stromal cells in a separate region of collagen surrounding the microvessels, Group 3 contained stromal cells mixed in with the microvessels, and Group 4 contained stromal cells both around and within microvessel containing regions [described in detail in Strobel et al. (2020)]. Phase contrast images were experiments comparing different microvessel donors or different culture medium types. The variation in treatment groups was intended to demonstrate the utility of the BioSegment software. The data presented and discussed in this manuscript pertains to the accuracy of BioSegment measurements, not the scientific findings of these experiments, which are already published (Strobel et al., 2020).

### Lectin Staining and Imaging

Constructs were fixed overnight in 10% neutral buffered formalin. Rat microvessels were further processed by staining with a fluorescently labeled lectin. After rinsing in phosphate buffered saline (PBS), constructs were permeabilized for 20 min in 0.25% Triton X-100 and blocked for 4 h at RT in 5% bovine serum albumin. They were incubated overnight at 4°C in lectin stain at a dilution of 1:50 in blocking solution [Griffonia (Bandeiraea) Simplicifolia Lectin I (GSL I, BSL I), Vector Laboratories]. Constructs were rinsed multiple times, with one overnight wash, before imaging. Human constructs were imaged using a phase contrast filter on a standard benchtop upright microscope (Olympus). Lectin-stained rat constructs were imaged with a confocal Olympus FV3000 or an INCell 6500 scanner (Cytiva, formerly GE Life Sciences), depending on the dataset. Confocal images were processed to create maximum z projections and saved as .png files before analysis.

### Application of the BioSegment Software

BioSegment utilizes YOLOv4, a machine learning deep convolutional neural network (CNN) to detect and localize user-defined classes within images (Girshick et al., 2014). It’s end-user facing front-end is desktop application written in C# and is used for image processing and annotation. Annotations and images are stored in the AWS cloud. Training is performed using AWS SageMaker. Trained models are retrieved from AWS cloud storage and transferred to the local storage for desktop inferencing via subordinate python process.

Prior to training, confocal images are transformed into a maximum projection if necessary and saved as a .png file using an ImageJ macro. After import into BioSegment, images can be further pre-processed through a histogram equalization to intensify features (Supplementary Figure 1). This can make the geometry easier for the algorithm to identify if the image has low contrast, and is done by selecting a “pre-process” option prior to training. Phase contrast images were converted to .png files and pre-processed with the same pre-processing function.

**FIGURE 1:**
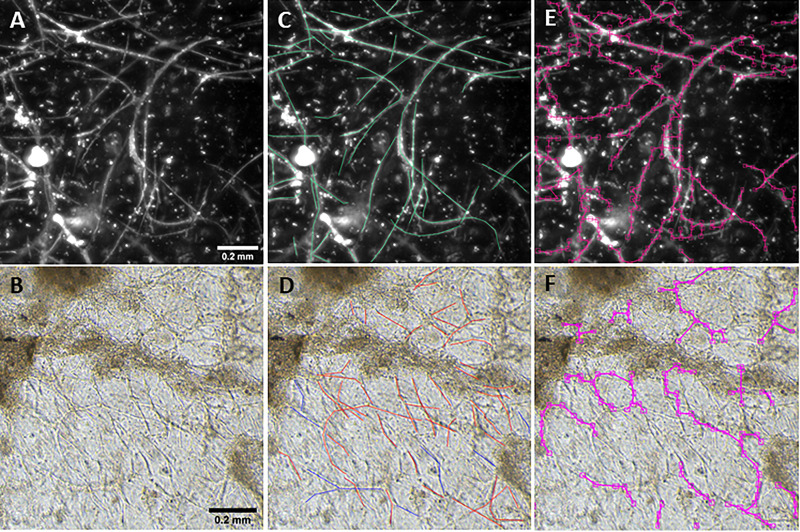
Vessel length density measurement. Fluorescent **(A)** and phase contrast **(B)** images taken at a 10× magnification. Annotations are manually added to images to mark vessels **(C,D)**. After training, the BioSegment software automatically identifies vessels and calculates vessel pixel length **(E,F)**, which is used to calculate vascular length density. Scale = 0.2 mm.

During implementation, a neural network was “trained” via expert annotation using polygonal chains (polylines) identifying user-defined features or classes of raw images within the BioSegment software environment (Figure 1). Polylines are generated by manually stepping over each vessel, and these lines are in turn bounded by a series of overlapping rectangles. These rectangles are the input regions for the model. Because a large number of rectangles are generated, YOLOv4 gains a large number (typically hundreds) of input regions from each image. Beyond this we utilized a set of pre-trained weights, thereby leveraging transfer learning to allow for generalization from a smaller data set. For the purposes of this application the YOLOv4-tiny.weights set of weights (trained on the MS-COCO dataset) were utilized.

When training is performed on a new dataset, training data is divided or “partitioned” into 3 sets (or groups): training, testing, and holdout. The “training partition” is the training group set for a specific fold. The “holdout” refers to the partition that was entirely segregated from the training process, to be used for later validation. After images were annotated (“train” data), the model was trained and then used to perform inference on new data (“test” data). With the BioSegment approach, images were annotated via the BioSegment interface by manually tracing each vessel to provide training sets for the machine learning engine, which then generated vessel measurements. For phase contrast images, the user also annotated “out of plane” vessels, which were too blurry for the user to tell if they were vessels or not, and “debris” (undigested pieces of tissue or other objects that are not microvessels). For confocal lectin-stained images, these extra parameters were not necessary, as confocal does not pick up out of plane objects, and the lectin stain will not label most debris.

**FIGURE 2:**
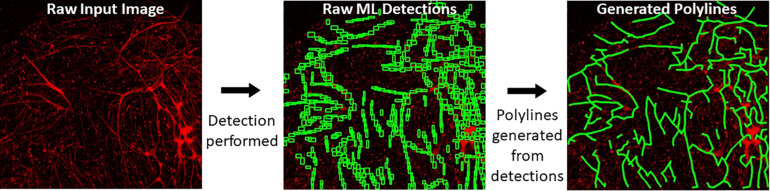
Polyline generation workflow. Raw images are subject to ML detections by YOLOv4. Then, BioSegment generates polylines from these detections.

**FIGURE 3:**
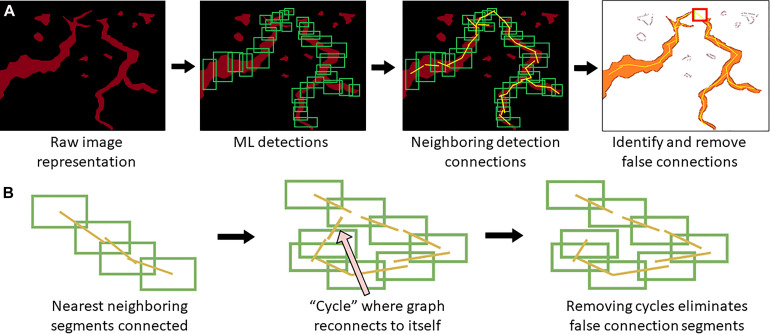
Polyline segmentation and cycle removal. A neighbor detection algorithm is used to create polylines from ML detections **(A)**. This can result in “false” connections between vessel segments, which are then removed using a spanning tree algorithm **(B)**.

When a trained model is used for object detection, YOLOv4 produces output detection regions (“raw ML detections”) which are processed into polylines through a BioSegment specific process (Figure 2). First, the object detection regions are connected using a neighbor detection algorithm. Then, a contour detection algorithm is used to eliminate “false” connections that are suggested by proximity, but do not represent actual vessels (Figure 3A). Finally, a minimum spanning tree algorithm removes cycles that are generated through the neighbor detection region and allowed by contour detection but not representative of vessel structures (Figure 3B). The most up to date version of BioSegment includes a feature that enables users to correct false segmentations, although this was not available at the time the present data was analyzed.

The BioSegment models were compared to two existing measurement protocols. First, we compared BioSegment data to manual tracings of the vessels, an approach widely considered the gold standard. Other programs for vascular quantification do exist, but these are designed for very specific applications. Our target users, who are scientists looking for quick assessments of vascular density in *in vitro* tissues, are still using manual annotations, by an overwhelming majority. Thus, manual annotations were our primary validation method. In addition, we compared the BioSegment measurements to our in-house pixel-based protocol in which images are processed to improve contrast, thresholded, filtered to remove small cells and large clumps, and skeletonized to measure vessel lengths (Strobel et al., 2020). The data reported are from either combined train and test datasets, or test-only datasets.

Total vessel length was calculated for each image and used to calculate vessel length density. Measurements from 4 images per sample were averaged to obtain the length density for each sample. Then, samples within each of the four experimental groups were averaged to determine the average vessel length density within each treatment group. These average vessel length densities, calculated using both measurement methods, was used to calculate percent error for each experimental group. Percent accuracy was calculated by subtracting percent error (below) from 100.

$$PercentError=PercentError=\frac{\left|Manuallyannotatedlength-BioSegmentcalculatedlength\right|}{Manuallyannotatedlengths}*100$$

Accuracy is reported for each group of each experiment, as different groups sometimes had different densities and morphologies, which affect overall accuracy. An accuracy of “N/A” is assigned to any group where the percent error was greater than 100.

### Machine Learning Methodology and Validation

The primary metric of concern related to vessel growth is vessel length density. Vessel lengths are measured manually by estimating the linear extents of visible vessels within a projected image (that is, one that is composed my merging images across a range of focal lengths), and then divided by image area to calculate density.

The approach presented here performs vessel length measurements through an application of object detection. The annotation process involves tracing the linear extents of vessels using polylines (joined line segments). Input object regions are programmatically constructed along the polylines and then used to train an YOLOv4 object detection model^1^. The input and output of object detection models such as YOLOv4 are rectangular bounding regions within a 2D image, which contain an object of interest along with bordering pixels. In most YOLOv4 applications, each input image within a training set will typically contain only 1 or a few examples of an object or objects which are the target of training, thus many images are required to produce enough input regions. In our case, there were often many microvessels in a given image. Thus, relatively small numbers of images (50–100 images) were used in a given training set.

The underlying model is an object-detection model and, as such, explanation algorithms such as Lime were not of value in performing feature importance/interpretability studies. Visual inspection was used to validate that both raw detections and constructed detection polylines represented vessels sufficiently well to produce valid linear extent measurements. This inspection showed that variance that is introduced by the process used to connect detection regions, that is, detection polylines have “kinks” or branches that would not be present in manual annotations, but such deviations tend to offset one another, and thus have a minimal impact on the final length measures.

A five-fold cross-validation was performed to evaluate the consistency of the vessel measurement algorithm. Two datasets were constructed using lectin-stained images at different magnification levels (referred to as “lectin 4×” and “lectin 10×”). The lectin 4× dataset was comprised of 108 total images from 35 samples. Samples were included from 4 experimental groups. The lectin 10× dataset was comprised of 24 samples, with 2–10 images per sample and 172 total images. Images within each dataset and within each image groups were assigned to training, validation, and holdout partitions. Training partitions were used to train each fold. Validation partitions were utilized by the training process to report training metrics. Image groups assigned to holdout partitions were entirely excluded from the data passed to the model during the training process. Error for a partition overall was calculated by averaging the percent error, as calculated above, for the partition. Overall, data was validated using 2 experiments for the lectin 10× model, 3 for the lectin 4× model, and 3 for phase contrast.

Overall, the cross-validation shows that the vessel-measurement algorithm sufficiently generalized across each fold such that any set of trained weights can be used reliably (Tables 1, 2). Error can be minimized by performing a cross-validation for a specific data set and selecting the best resultant weights for detection.

**TABLE 1:** Cross validation results for the model trained to analyze 4× lectin images.

**Fold**	**Partition**	**% Error**
**Lectin**		
1	holdout	0.0845
1	validation	0.5605
1	train	2.7385
2	holdout	5.8329
2	validation	2.1858
2	train	4.619
3	holdout	12.8576
3	validation	7.2046
3	train	2.8441
4	holdout	0.8912
4	validation	3.6903
4	train	0.4749
5	holdout	1.5407
5	validation	5.5789
5	train	0.4428

**TABLE 2:** Cross validation results for the model trained to analyze 10× lectin images.

**Fold**	**Partition**	**% Error**
**Lectin 10×**		
1	holdout	19.351
1	validation	11.3737
1	train	16.1774
2	holdout	11.0625
2	validation	15.2432
2	train	15.6101
3	holdout	13.4747
3	validation	14.6666
3	train	16.2374
4	holdout	14.8235
4	validation	11.2876
4	train	16.1631
5	holdout	23.9069
5	validation	16.7967
5	train	12.9337

## Results

### BioSegment Accurately Measured Multiple Types of Images

**FIGURE 4:**
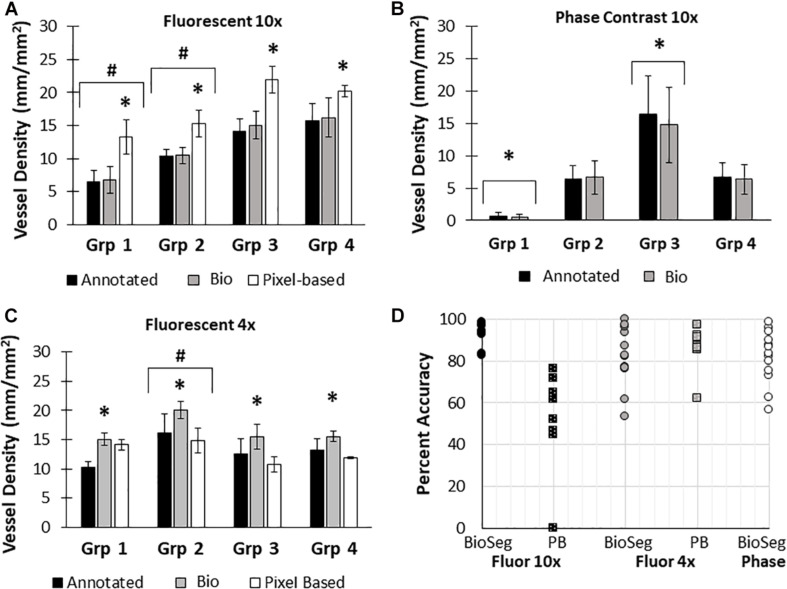
Accuracy of BioSegment vessel measurements. BioSegment calculated vessel length densities (Bio) were compared to manual annotations (Annotated) and pixel-based (PB) measurements. Representative datasets are shown for 10× fluorescent confocal projections **(A)**, phase contrast images **(B)**, and 4× confocal stacks **(C)**. The percent accuracy of the BioSegment (circles) and PB (squares) compared to manual annotations is shown in **(D)**, across all groups from all experiments analyzed. Bars are mean ± SD. A Two-Way ANOVA with Holm Sidak *post hoc* analysis was performed on **(A–C)**. ^∗^*P* < 0.05 with compared to all other quantification methods (Annotated, Bio 1, PB) within that group; ^#^*P* < 0.05 compared to all other groups, regardless of quantification method.

Here, we created a program that leverages artificial intelligence and machine learning to identify tissue components, specifically microvessels. The interface was, subjectively, easy to use, and enabled users to upload and annotate images needed for training. Figure 1 shows images of fluorescent (A) and phase contrast (B) images prior to processing. After manual annotation (C, D), the model is trained and then “learns” to identify vessels (E, F). Representative datasets show vessel length density as calculated by manual annotation, our previously published pixel-based method, and by BioSegment (Figure 4). The percent accuracy was calculated for each group within each dataset, as BioSegment compared to either manual annotations or the pixel-based method (Figure 4D). The fluorescent 10× images had the highest accuracies, with percentages ranging from 82.79 to 98.74% accurate. Most accuracies were above 90%. On the same images, the pixel-based measurements had an accuracy of 0–76%, though all but one point was above 44%. With lower magnification images (4×), BioSegment had accuracies of 53.4–99.74% (Figure 4D). In these images, subjectively, the lower accuracies occur in images with the highest vessel densities. For the 4× images, pixel-based accuracies were comparable to BioSegment, ranging from 62.2 to 96.95% (Figure 4D). For phase contrast images, which were taken at a 10× magnification, accuracy ranged from 56.4 to 98.48% (Figure 4D).

### Accuracy of BioSegment Improved With Time

**FIGURE 5:**
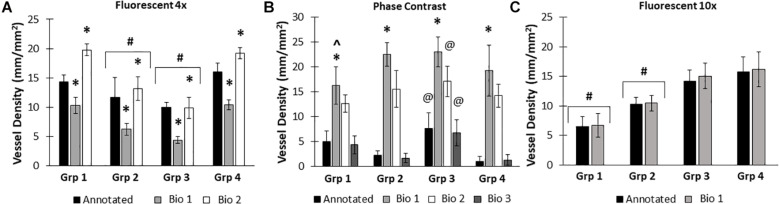
Increasing the number of training datasets improves model accuracy. Representative datasets for 4× fluorescent images **(A)** and phase contrast images **(B)** after each iteration of the respective model. Annotated images are compared to multiple versions of the BioSegment model (Bio 1, Bio 2, Bio 3). Later versions, which contain more training sets, are more accurate. In (A), additional training data increased overall accuracy from 58.5 to 82.13%. In **(B)**, Bio 3 (model version 3) improved to 83% accurate, compared to 0% in Bio 1 and Bio 2 (model versions 1 and 2). Bars are mean ± SD. A Two-Way ANOVA with Holm Sidak post hoc analysis was performed. *P < 0.05 compared to all other quantification methods (Annotated, Bio 1, Bio 2, Bio 3) within that group; ^#^*P* < 0.05 compared to all other groups, regardless of quantification method. ^@^*P* < 0.05 compared to groups 2 and 4, within that quantification method. ^*P* < 0.05 compared to other methods within that group.

Due to differences in vascular morphology from experiment to experiment, sometimes a model trained on a dataset from a single experiment failed to yield satisfactory results when tested on a different experiment. In these cases, another training set, from an additional experiment, was added, to add more variety in terms of vascular morphology to the model. After multiple training sets, the model demonstrated increased accuracy across subsequent datasets from multiple experiments, regardless of vascular morphology. Accuracies improved after 2 training sets for the 4× images (81 images total, Figure 5A). Here, black bars are annotated controls, gray bars are the BioSegment output after one round of training (Bio 1), and white bars are after 2 rounds of training (Bio 2). In groups 2, 3, and 4, one round of training resulted in inaccurate measurements compared to annotated controls. When a second training dataset was added, accuracy was improved and white BioSegment bars are similar in magnitude to black annotation bars. The exception to this is group one, where both rounds had different magnitudes compared to controls. For phase contrast images, 3 different training datasets were needed (155 images total; Figure 5B). Here, the software was highly accurate after a third round of training across all groups (dark gray bars compared to black bars). The 10× fluorescent images had a high accuracy after one large training set (79 images; Figure 5C). Statistics were performed using SigmaPlot 11.0 (Systat). A two-way ANOVA test was performed where applicable with Holm-Sidak *post hoc* analysis. Bars are mean ± standard deviation. A significant level of α = 0.05 was used for all comparisons to determine statistical significance.

### Use of BioSegment Saves Considerable Amounts of Time

It took an experienced scientist 7.26, 4.33, and 2.78 min to annotate a single 4× fluorescence, 10× fluorescence, and 10× phase contrast images (averaged over 3 images of varying density), respectively. The pixel-based method takes an experienced user approximately 80 s per image. Meanwhile, the BioSegment analyzed 45 images in 52 s, for an average of 1.15 s per image. For a single dataset of 45 images, the BioSegment therefore saves from 124 to 325 min of time (2–5.4 h), compared to manual annotations.

### “Training and Test” Combined Data Are Comparable to “Test Only” Data

**FIGURE 6:**
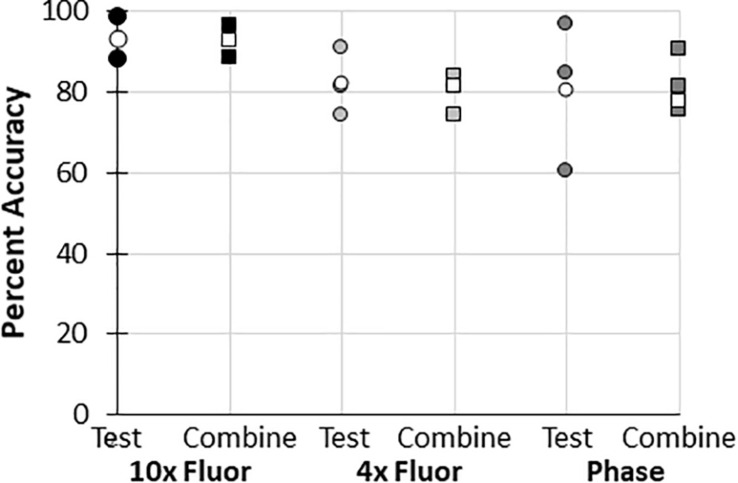
Accuracy of “test and train” combined data compared to “test only” data. Percent accuracy of the BioSegment compared to manual annotations is shown for image sets containing both test and train images (combined; squares), compared to test only images (test; circles) from the same dataset. Individual experiments are shaded, while the average of all experiments is shown in white.

In all datasets used to train the BioSegment models, some images were used for training, while some were used to “test” the model. Whenever possible, the data is reported using “test” images only (Figure 4C, and 2 additional experiments plotted in Figure 4D). However, in some cases, data is presented as a combination of the training and test images in a dataset, to enable comparison of multiple groups within an experiment, as there were not quite enough “test” samples for meaningful comparisons (Figures 4A,B, and 3 additional experiments plotted in Figure 4D). To test the validity of the combined test and training data, the accuracy of combined data (compared to manual annotations) was compared to the “test only” images pulled from the same combined dataset. In all cases, average accuracy measurements of test only vs. combined test and train were within 2 percentage points (Figure 6). Thus, we can conclude that including training images in our datasets did not skew the calculated accuracy of the results.

## Discussion

A variety of applications benefit from or require quantitative assessments of vascularity. Whether in an experimental model of angiogenesis or vascularizing a tissue construct or organoid, knowing the extent and character of the vascular dynamics can be critical to successful outcomes. Furthermore, as tissue fabrication solutions continue to evolve matched by concomitant development of tissue manufacturing processes, real-time assessments of vascularity (perfused or otherwise) are critical. Yet, most methods of quantifying vascular growth are challenging to use without prior experience in computer programming, not conducive to high-throughput applications, or cannot accurately detect complex neovasculatures. Here, we describe a novel machine learning-based software program for semi-automatic quantification of microvessel length density that is simple to use yet leverages the power of machine learning. While directed at assessing vascularity, reflecting our research focus, BioSegment could be adapted to identify other cellular and tissue features as well. In addition to facilitating research investigations, we envision integrating BioSegment as part of a quality assurance program in a tissue manufacturing workflow.

Here, we compared measurements of vessel length density made by BioSegment to manual annotations made “by hand,” and a pixel-based computational method previously developed by our lab. The BioSegment-based measurements of vessel length density from 10× fluorescent confocal images were overall highly accurate compared to manual assessments, both in overall vessel length density magnitude and the trends measured between groups (Figure 4). On the same images, the pixel-based approach was considerably less accurate (Figure 4). Pixel-based measurements tended to overestimate vessel density. This is because it cannot distinguish between microvessels and other objects with similar dimensions to microvessels, such as elongated endothelial cells and some pieces of undigested tissue. Both 4× fluorescent confocal images and 10× phase contrast images produced a broader range of accuracies across the different treatment groups, although most were above 80% accurate (Figure 4D). With 4× fluorescent images, the accuracy of both the pixel-based and BioSegment approaches both had slightly lower reported accuracies (Figure 4D). The pixel-based approach was not able to derive vascular length density measurements from phase images due to the lower contrast. In all cases, data produced by the BioSegment within each experiment followed the same trends as annotated controls. For example, in Figure 4C Group 2 was highest regardless of quantification method. Thus, even though there is a small degree of error in the magnitude, the user is able to draw the same conclusions. Additionally, while this 20% error seems high, it is comparable to what others have observed in cases where machine learning was applied to biological systems (Liang et al., 2017; Tourlomousis et al., 2019). The observed error may improve with additional training, as is true of all machine-learning based applications. With our data, we observed that more training datasets improved performance (Figure 5). It should also be noted that, images contained a substantial amount of noise in the form of non-vessel elements, such as elongated endothelial cells and clumps of undigested tissue. Additionally, there were slight differences in vascular morphology from experiment to experiment. The reported accuracies reflect the ability of BioSegment to distinguish microvessels from this other noise, which further supports the robustness of the program. Cleaner images would likely produce better results.

It was not surprising that the higher magnification images yielded a high percent accuracy, as individual vessel features are more discreet, thereby making it easier both for the user to annotate and BioSegment to segment. For the low magnification confocal images, and particularly those with dense vasculatures, identifying individual vessel segments by both the user and the BioSegment model is relatively challenging. This is largely due to non-distinct boundaries between two or more vessel segments and the intrinsic variation in fluorescence intensities of different vessels, all of which are highly overlapping in the projections. In the future, magnifying images for annotating, in the absence of taking higher magnification images, may improve user annotations and thus BioSegment training. Additionally, extending the analysis to work with 3D image sets, as opposed to the 2D projections used in this study, might facilitate identification of discreet features. Future iterations of BioSegment will include the ability to measure vascular density and other parameters, such as branch points, directly from a 3D confocal image stack. This would enable measurements in Z, as well as X and Y. However, the ability to assess 2D projections makes the program more amenable to rapid, high-throughput screening. From past experience, microvessels in our models grow largely in the X and Y plane, rather than in the Z direction (due to collagen fibril orientation). Thus, the amount of data lost by eliminating growth in Z is minimal and the ability to analyze 3D confocal stacks is not necessary for our current application. Accuracies were determined by comparing BioSegment and pixel-based measurements to those values obtained by manual annotation, which we consider the “gold standard.” However, measurements made by manual annotations, even by the most experienced user, can be inaccurate for a variety of reasons (Rudyanto et al., 2014), including subconscious bias, mood, etc. As mentioned above, overlapping vessels, and varying fluorescent intensities, can make it challenging to accurately annotate. Phase contrast images are even more challenging to annotate, as contrast between feature edges and background is often low creating ambiguity in object identification. If the software is trained with inaccurately annotated training sets, it may also have reduced accuracy. One potential way to overcome this challenge is to include training sets from multiple users, which may negate potential user bias and differences in mood/attentive states. Providing sufficient training to the BioSegment model in this way could potentially make it more accurate than a single human user. Furthermore, the ability to identify features from phase contrast images, which can be taken of live cultures quickly and easily without the risks associated with staining, presents an enormous advantage, even if there is some risk of quantitative error.

The amount of time saved by using the BioSegment was considerable, with potential to save users hours of time per experiment. This effect is magnified if users have multiple datasets. The rapid measurements possible with the BioSegment platform have implications for use in high throughput and high content screens and cell/tissue manufacturing efforts. Here, there may be hundreds of samples in culture that require imaging at frequent time points to assess tissue quality and maturation. This could generate thousands of images, which will be impossible to quantify without automated analysis.

Overall, we have demonstrated an innovative software system that utilizes machine learning to quantify microvessel length. It can rapidly and accurately measure vascular features from both fluorescent and phase contrast images. Such a tool saves users an enormous amount of time and has potential to be incorporated into automated processes such as assay screens or quality control in tissue manufacturing. While we focused on training the program to identify microvessels, it could potentially be used to identify any tissue element, including but not limited to cellular density, subcellular features, or contaminants. This could be done following the same procedures for identifying microvessels. The ability to make varied, accurate, and rapid measurements via a customizable and flexible package may prove invaluable for automation of tissue fabrication, quality control, and real-time monitoring in an automated workflow.

## Data Availability Statement

The raw data supporting the conclusions of this article will be made available by the authors, without undue reservation.

## Ethics Statement

The animal study was reviewed and approved by the IACUC Committee at Dartmouth College.

## Author Contributions

AS wrote and edited the coding. HS collected the data, validated the software, and drafted the manuscript. JH and RE supervised code development and experimental design. All authors contributed to the initial conception, development of BioSegment, and edited the manuscript.

## Conflict of Interest

JH and RE are partners of Advanced Solutions. All authors are employees of Advanced Solutions.

## Footnotes

1 https://github.com/AlexeyAB/darknet

## Supplementary Material

Supplementary Figure 1 Histogram equalization. Example images before **(A)** and after **(B)** histogram equalization. The original histogram **(C)** has clip points identified using a defined threshold **(D)** to create a rescaled distribution **(E)**. Blue represents original histogram, and orange represents the new histogram.
