# Supplementary figures and images for: Quantifying Vascular Density in Tissue Engineered Constructs Using Machine Learning
Source: Front Physiol. 2021 Apr 27;12:650714. doi: 10.3389/fphys.2021.650714 (PMC8110917; doi:10.3389/fphys.2021.650714)

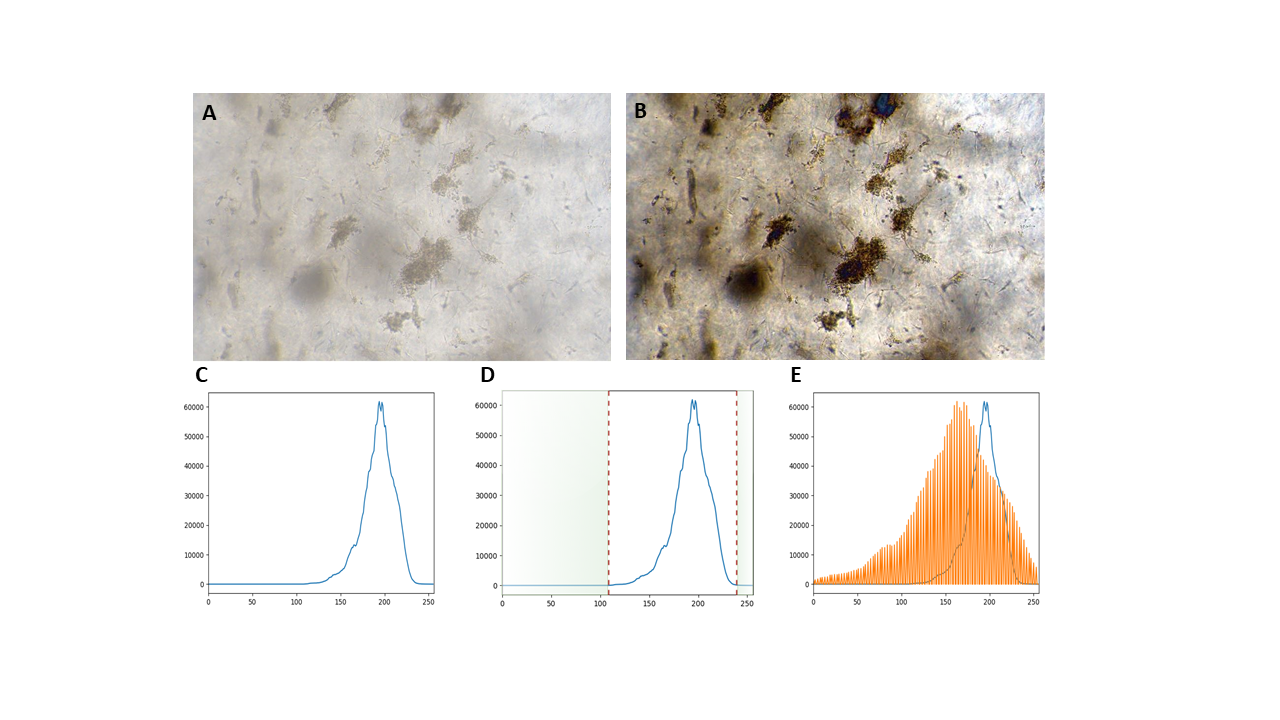

Supplement: Supplementary Figure 1 — Histogram equalization. Example images before (A) and after (B) histogram equalization. The original histogram (C) has clip points identified using a defined threshold (D) to create a rescaled distribution (E). Blue represents original histogram, and orange represents the new histogram. [file Image_1.TIF]
